# Needs Analysis for Non-Face-to-Face Services among Older Adults to Reduce Loneliness

**DOI:** 10.3390/healthcare10081576

**Published:** 2022-08-19

**Authors:** Hee Kyung Choi, Seon Heui Lee

**Affiliations:** Department of Nursing Science, College of Nursing, Gachon University, Incheon 21936, Korea

**Keywords:** aging, community, loneliness, non-face-to-face, information and communication technology, needs assessment

## Abstract

Background: Considering older adults’ interests and physical abilities, non-face-to-face services precipitated positive acceptance and reduced loneliness. Developing non-face-to-face services designed after investigating older adults’ needs is necessary. Research question: What is the need for non-face-to-face services to reduce loneliness among older adults in the community? Methods: A questionnaire was developed through a content validity evaluation of a group of experts based on a prior systematic review. The survey was administered to 100 community-dwelling older adults between 15 July and 31 August 2021. The need for non-face-to-face services for older adults was analyzed. Results: This study found that non-face-to-face services precipitate positive perceptions and satisfy the needs of older adults in the community. Additionally, the older adults preferred integrated content from non-face-to-face services. Through the analysis of preference differences according to the general characteristics, this study shows the possibility of inducing participation by developing content that attracts older adults’ attention. Conclusion: This study is expected to provide basic data for content development of non-face-to-face services to reduce loneliness among older adults in the community by investigating their needs.

## 1. Introduction

Old age is a stage of life with a high risk of exposure to disease, poverty, incompetence, and solitude due to aging; additional life events related to loss due to the death of a loved one [1] further increase loneliness among older adults [2]. Loneliness affects older adults’ health and life satisfaction, and causes them to experience a high level of depression and cognitive decline [3]. This situation requires strong consideration and demands an intervention strategy.

Although face-to-face services are operated for community-dwelling older adults, efficiently utilizing resources is difficult and exhibits limitations [2,4,5,6]. Previous studies have focused on the need for non-face-to-face services to improve social participation and health by reducing loneliness among older adults in the community [5,6,7]. Additionally, the prolonged COVID-19 pandemic has required the utilization of information and communication technology (ICT) to continuously provide non-face-to-face services rather than direct contact [8]. Kim [9] reported that the quality of care services can be improved using science and technology. Therefore, developing non-face-to-face services is needed to reduce community dwelling older adults’ loneliness.

To reduce older adults’ loneliness, various non-face-to-face services—despite becoming increasingly important—have not been properly utilized [10]. Moreover, older adults prefer utilizing easy-to-use and practically helpful non-face-to-face services [5,6,7]. Further, a previous study reported that considering older adults’ interests and physical abilities, non-face-to-face services were positively accepted and reduced loneliness [6]. Therefore, developing non-face-to-face services designed for older adults by investigating their needs is vital.

**Figure 1:**
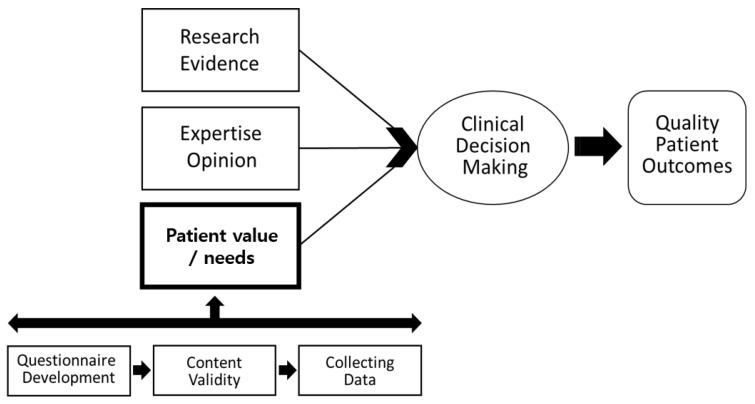
Research frame work [11].

In this regard, evidence-based practice (EBP)—an effective problem-solving approach that integrates optimal evidence from relevant research, expert opinion, and patient needs, to build non-face-to-face services to reduce loneliness in older adults—is worth consideration (Figure 1) [11]. A great deal of evidence already suggests that non-face-to-face services positively affect the reduction of loneliness among older adults [2,6,12].

Previous studies on non-face-to-face services for older adults only identified the attitude and intention to use silver care robot technology [13] and the experience level in using smart healthcare [14,15,16]; however, research on older adults’ needs is limited. A previous study [17] aimed to confirm the perception, preference, and acceptance of ICT among older adults, but only 15 subjects were included in an isolated area, thereby making it difficult to represent the entire population of older adults. These previous studies have been controversial and inclusive of older adults’ attitude toward non-face-to-face services and have not analyzed the need for specific content. Therefore, the purpose of this study was to investigate the perceptions, needs, and preferences of non-face-to-face services for older adults to reduce loneliness based on questionnaires developed by scientific research evidence and experts.

## 2. Materials and Methods

This study is a cross-sectional study that analyzes the perceptions, needs, and preferences of community-dwelling older adults and develops content on non-face-to-face services to reduce loneliness of older adults.

### 2.1. Research Frame Work

Evidence-based practice (EBP), a problem-solving approach through a clinical decision-making process that integrates research evidence, expert opinion, and patient needs, improves the quality of patient care (Figure 1) [11]. As for the patient’s needs, data are collected through surveys after evaluating the content validity of the developed questionnaire. This study investigates the perceptions, needs, and preferences of non-face-to-face services for older adults based on the questionnaire developed by scientific research evidence and experts.

### 2.2. Participants and Sample Size Estimation

The study included participants who fulfilled the following criteria: (1) adults aged 65 years or older, (2) residing in local communities, and (3) no cognitive impairment. Those unable to complete the survey—such as those with cognitive impairment or who had difficulty communicating—were excluded.

The sample size was calculated using the G-Power 3.1. Based on Cohen [18], the sample size was calculated with an alpha error of 0.05, power of 80%, and an effect size of 0.15 (F-test, linear multiple regression: fixed model, number of predictors = 5); therefore, a sample size of 92 participants was required. After considering the dropout rate, a total of 100 participants were recruited.

### 2.3. Questionnaire Development and Content Validity

We developed a questionnaire to investigate the need for non-face-to-face services for older adults through the following process: Several meetings were conducted between researchers, and a prior systematic review was referred to [12]. Thereafter, a group of experts comprising three nursing professors evaluated the draft on a four-point scale, and the content validity index (CVI) was calculated. The CVI score was 0.99 for content validity and 1.00 for importance. One question with a CVI score of 0.67 was revised to a more specific phrase following the experts’ opinion. The final questionnaire included (1) respondents’ general characteristics, (2) perceptions and necessity for non-face-to-face services, and (3) non-face-to-face services’ preferred components. The questionnaire is provided in Appendix A.

### 2.4. Collecting Data and Statistical Analysis

We consecutively recruited 100 older adults visiting 7 senior citizen centers—including urban and rural areas in the Korean community—from 15 July to 31 August 2021. The researchers explained the purpose and contents of the study to older adults who met the selection and exclusion criteria of the study among older adults in the senior citizen centers, obtained written consent, and conducted a one-on-one survey for 15 to 20 min. The 100 questionnaires were collected and used for the analysis.

The analysis predominantly focused on two aspects: the older adults’ perception and necessity of non-face-to-face services and preference for components of the non-face-to-face services. The collected data were analyzed by frequency, percentage, mean, and standard deviation (SD) for the participants’ general characteristics, perceptions, necessity, and preferences for non-face-to-face services—using the SPSS WIN 26.0 program (Version 26.0, IBM Corp., Armonk, NY, USA). The difference in participants’ preference for non-face-to-face services—according to their general characteristics—was analyzed using the independent *t*-test, Mann–Whitney test, and Kruskal–Wallis test, following the normality test.

### 2.5. Ethics

This study was approved by the Institutional Review Board of Gachon University in Incheon, South Korea (IRB No. 1044396-202102-HR-019-02). Prior to administering the survey, the study’s purpose and contents were explained to the participants, who then signed a consent form.

## 3. Results

In this part, we analyzed participants’ general characteristics, perceptions, and necessity of non-face-to-face service, preferred components of non-face-to-face service, and differences in preference related to non-face-to-face services according to participants’ general characteristics.

### 3.1. Participants’ General Characteristics

The general characteristics of the study participants included in the survey were analyzed (Table 1). The survey was administered to 100 older adults aged 65 years or older living in the community between July and August 2021; all 100 questionnaires were analyzed. Before commencing the survey, the researcher explained to the participants that the non-face-to-face service is to mediate older adults’ health, safety, and emotional state with electronic devices—such as smartphones and tablets—without meeting people. The participants’ mean age was 73.2 ± 7.0 years old, 67 (67%) of which were women. Of the total participants, 63% had “high school or higher” education, 67% were in the “middle” in self-rated economic status, and 66% were in the “middle” in self-rated health status. When asked if they exercise, “yes” (71%) was the majority response; regarding the types of exercise, there were multiple responses, including “walking” (81.7%) and “gymnastics” (15.5%). Further, 52 respondents (52%) stated that they had previously used smart devices.

### 3.2. Perceptions and Necessity of Non-Face-to-Face Services

We analyzed perceptions and necessity of non-face-to-face services for older adults (Table 2). Participants responded to the need for non-face-to-face services as follows: 7% “Strongly agree”, 41% “Agree”, 29% “Weakly agree”, 12% “Neither agree nor disagree”, 8% “Disagree”, and 3% “Strongly disagree”. Regarding the intention to use non-face-to-face services, 72% answered “yes”, while 28% answered “no”. Thus, most older adults seem willing to use non-face-to-face services. The number of responses regarding the appropriate frequency of non-face-to-face services was 38% for “twice a week” followed by 37% for “once a week”. Regarding the most suitable amount of usage time, 47% responded “less than 30 min”, followed by 44% who preferred “more than 30 min and less than an hour”.

### 3.3. Preferred Components of Non-Face-to-Face Service

Preferred components of non-face-to-face services for older adults were analyzed (Table 3). Participants were asked to score their preference for non-face-to-face service components on a 5-point scale. Most responses were positive with more than three points, including “Health assessment” (3.67 ± 1.17), “Sharing pictures” (3.41 ± 1.25), and “Listening to music” (3.37 ± 1.15). However, preference was low, in order, for “Daily intake record” (2.78 ± 1.13) and “Silver game” (2.90 ± 1.33).

### 3.4. Differences in Preference Related to Non-Face-to-Face Services According to Participants’ General Characteristics

The participant’s differences in preferences for non-face-to-face service contents according to their general characteristics—such as age, gender, education, smart device experience, exercise implementation, self-rated health status, and self-rated economic status—were analyzed.

#### 3.4.1. Differences in Preference Related to Non-Face-to-Face Services According to Age

We analyzed the differences in preference related to non-face-to-face services according to age of participants (Table 4). The Kruskal–Wallis test was performed after the normality test to detect differences in content preference according to age. The mean rank of preference for “Health assessment” was 44.73 for those under 70 years old, 60.34 for those aged 71–80, and 48.79 for those over 81 years old (*p* = 0.045). The post-hoc analysis using the Mann–Whitney test revealed that the mean rank of health assessment preference was higher for those aged 71 to 80 years than for those under 70 years of age.

#### 3.4.2. Differences in Preference Related to Non-Face-to-Face Services According to Gender

The difference in preference related to non-face-to-face services according to the gender of the participants was analyzed (Table 4). To analyze content preference according to gender, we performed an independent *t*-test after the normality test. The mean and standard deviation of preference for “Listening to the music” was 2.97 ± 1.10 for men and 3.58 ± 1.12 for women, showing a statistically significant difference (*t* = 2.54, *p* = 0.013). The preference for “Sharing pictures” was 2.85 ± 1.06 for men and 3.69 ± 1.25 for women, showing a statistically significant difference (*t* = −3.31, *p* = 0.001).

#### 3.4.3. Differences in Preference Related to Non-Face-to-Face Services According to Education

The researcher analyzed the difference in preference for non-face-to-face services according to education (Table 4). After the normality test, an independent *t*-test was performed to analyze content preference according to the educational level. The mean preference for “Nutrition education” was 2.73 ± 1.28 for middle school or lower, and 3.32 ± 1.28 for high school graduates or higher, indicating a statistically significant difference (*t* = 2.21, *p* = 0.029).

#### 3.4.4. Differences in Preference Related to Non-Face-to-Face Services According to Smart Device Experience

The difference in preference related to non-face-to-face services according to smart device experience was analyzed (Table 5). After the normality test, an independent *t*-test was performed to analyze content preference according to smart device experience. The mean preference for “Walking” was 3.56 ± 1.13 with experience and 2.96 ± 1.10 for no experience, showing a statistically significant difference (*t* = 2.67, *p* = 0.009). The mean “Health assessment” preference was 3.92 ± 1.22 with experience and 3.38 ± 1.05 with no experience, showing a statistically significant difference (*t* = 2.36, *p* = 0.020). The mean “Daily intake record” preference was 3.04 ± 1.13 with experience and 2.49 ± 1.08 with no experience, indicating a statistically significant difference (*t* = 2.48, *p* = 0.015). The mean preference for “Life information” was 3.60 ± 1.26 with experience and 3.02 ± 1.26 with no experience, indicating a statistically significant difference (*t* = 2.31, *p* = 0.023).

#### 3.4.5. Differences in Preference Related to Non-Face-to-Face Services According to Exercise Implementation

We analyzed the difference in preference related to non-face-to-face services according to exercise implementation (Table 5). For the analysis of content preference according to exercise implementation, the Mann–Whitney test was performed after the normality test. Comparing the mean rank of content preference revealed significance differences in “Daily intake record” (Z = −2.51, *p* = 0.012) and “Nutrition education” (Z = −2.49, *p* = 0.013).

#### 3.4.6. Differences in Preference Related to Non-Face-to-Face Services According to Self-Rated Health Status

The difference in preference related to non-face-to-face services according to self-rated health status was analyzed (Table 6). The Kruskal–Wallis test was performed after the normality test to analyze content preference according to self-rated health status. The mean ranks of preference for “Listening to music” were 38.10, High; 54.05, Middle; and 45.53, Low (*p* = 0.098). The mean ranks of preference for “Sharing pictures” were 37.53, 50.70, and 60.03, respectively (*p* = 0.070).

#### 3.4.7. Differences in Preference Related to Non-Face-to-Face Services According to Self-Rated Economic Status

The researcher analyzed the difference in preference for non-face-to-face services according to self-rated economic status (Table 6). Content preferences according to self-rated economic status were analyzed using the Kruskal–Wallis test after the normality test. No statistically significant differences were found in the content preferences’ mean rank.

## 4. Discussion

With this study, we aimed to examine the needs of non-face-to-face services for community-dwelling older adults to reduce loneliness. In this section, we discuss the results by dividing them into perceptions and necessity, preferred component, and differences in preference according to the general characteristics of the participants. This survey indicates that non-face-to-face service is necessary for older adults.

### 4.1. Perceptions and Necessity

We discussed perceptions and necessity of non-face-to-face services for older adults. In this survey, 7% “Strongly agree”, 41% “Agree”, and 29% “Weakly agree” answered that they needed non-face-to-face services, while 72% stated that they were willing to use these services. These findings correspond to the trend of older adults’ attitude toward ICT gradually changing to positive, as shown in past studies [19]. Furthermore, in a previous study, reliable technology and perceived pleasure reinforced attitudes and intentions to use silver-care robot technology [13]. Reportedly, older adults greatly appreciate technology’s reliability [20], and enjoyment becomes an important motivating factor affecting intention to use [21]. Therefore, considering technologies’ reliability and entertainment functions, the development of non-face-to-face service content is expected to strengthen older adults’ positive attitudes and high usage intention.

The number of interventions and time per session for non-face-to-face services required by older adults were discussed. The participants stated that “twice a week” (38%) was appropriate for the number of interventions, and preferred “less than 30 min” (47%) per session. In a previous systematic review [12], the number and time of interventions varied per intervention, but this is similar to previous studies that conducted ICT interventions twice a week for older adults [22,23]. Additionally, this finding is similar to previous studies’ results that an interaction of about 30 min must be maintained to evaluate the interactivity of the older adults who live independently [24,25]. Therefore, if non-face-to-face services are organized considering the number of interventions and time per session, older adults’ participation can be effectively improved.

### 4.2. Preferred Components

We discussed the preferred components of non-face-to-face services for older adults by dividing them into less than 3 points and more than 3 points. The majority of preference for non-face-to-face services content was greater than three points, and we confirmed that the subjects desired integrated intervention. This is similar to a previous systematic review’s finding that most studies utilized integrated interventions [12]. Therefore, developing integrated non-face-to-face services for older adults is required. On the other hand, a preference of less than 3 points was confirmed for “Talking with smart devices,” “Silver game,” “Gymnastics with music,” and “Daily intake record.” Arguably, this is because the related repeated and tedious inputs involve psychological burdens and reduce their interest. In a previous study, enjoyment was high when non-face-to-face services were developed for older adults [6]. Therefore, designing entertaining content, which is easy to use, is necessary.

The results of the importance of reflecting the preferences of older adults in the development of non-face-to-face services were discussed. Our study investigated the activities preferred by older adults in terms of songs, games, books, and exercises; reflecting these results in service content is crucial. Previous studies have confirmed emotional and cognitive effects by investigating older adults’ favorite genre of songs and books in program organization [22,26]. Moreover, most respondents indicated that games are fun leisure activities and expressed a desire to learn sports through games [27]. Therefore, to induce interest in older adults and motivate them to participate in non-face-to-face services requires incorporating the preferences found in the survey into the services.

### 4.3. Difference in Preference According to General Characteristics of Participants

The results of the differences in preferences for non-face-to-face services according to the general characteristics of the participants were discussed. There was a significant difference in preference for non-face-to-face service content by age, gender, education, smart device experience, exercise implementation, and self-rated health status; however, there was no difference related to self-rated economic status. Analyzing preference differences according to the participants’ general characteristics revealed that such services are expected to be effective for the community older adults.

The preference for “Health assessment” was significantly higher in those aged 71 to 80 years compared to those aged under 70 and over 81. This resembles the results of a study that showed that 70–74-year-olds exhibited a high ability to understand health information [28]. A previous study reported that the lower the age of the older adult, the higher the level of informatization [14]. This is consistent with the higher mean rank (though not statistically significant) of preference (60.34 vs. 48.79%) in our study. However, the lack of interest in “Health assessment” among older adults under the age of 70 is due to older adults’ increasing population and life expectancy; the related mean age subjectively perceived by the older adults was 71.4 years [29]. Here, the older adults under the age of 70 do not recognize themselves as older adults, which can be interpreted as showing limited interest in non-face-to-face services.

The preference for “Listening to music” and “Sharing pictures” was significantly higher in women than in men. These results differ from previous studies wherein men exhibit relatively high information ability and high Internet and mobile use [15], but are similar to those of studies wherein older women exhibit a higher preference for cultural and artistic activities that improve life satisfaction [30]. If this study’s results are reflected in the development of non-face-to-face services, this would increase older adults’ participation.

The preference for “Nutrition education” was significantly higher for participants with a high school education or higher than for those with middle school education or lower. This is similar to prior results wherein the higher the educational level, the greater the understanding of health information [28]. Therefore, older adults with a higher level of education are expected to improve their ability to utilize the services required for healthcare using non-face-to-face services.

In the case of using smart devices, the preferences for “Walking,” “Health assessment,” “Daily intake record,” and “Life information” were significantly higher. This resembles the results of a previous study wherein smart devices were used to access mass media information [16]. Therefore, older adults with experience using smart devices are expected to exhibit a greater inclination to use the content providing useful information.

Participants who responded that they exercised exhibited a significantly higher preference for “Daily intake record” and “Nutrition education.” This is similar to a previous study’s results that people are interested in and participate in health care in their daily lives using smartphones when they exercise regularly [31]; older adults who exercise are, thus, expected to exhibit a high intention to use the service.

In terms of content preference based on self-rated health status, older adults’ preference tended to be significantly higher for “Listening to the music” and “Sharing pictures,” but there was no statistically significant difference for other content. In this study, a small number of participants were classified by health conditions; therefore, an analysis based on a sufficient number of participants is required in the future.

There was no difference in content preferences based on self-rated economic status. This is different from previous studies’ results that the difference in Internet usage ability and use frequency depend on economic conditions and low smartphone usage rate [15]. However, owing to the advent of smart mobile environments, all participants here had an environment wherein they could use smart devices, and most respondents stated that they used smart devices, assumedly owing to high accessibility. In this study, 100 older adults were analyzed, but accurately representing the difference in preferences—according to economic status—is difficult due to the small number of participants.

### 4.4. Limitations

This study has certain limitations. First, the analysis’ results are diverse, and representing the entire older adult population is difficult due to the small number of survey respondents. In future studies, calculating and analyzing a greater number of participants will be necessary. Second, in terms of the need for non-face-to-face services, the response options may not be neutral for response analysis because it is not a formula Likert style.

## 5. Conclusions

This study investigated older adults’ needs, using their responses as basic data for developing non-face-to-face service content to reduce loneliness among older adults in the community. This study confirmed the need and intention to use non-face-to-face services among older adults. Further, we confirmed that older adults prefer the majority of non-face-to-face service content. This study is expected to attract the attention of older adults and encourage their participation in the development of non-face-to-face services.

Based on our study, developing non-face-to-face services is expected to enhance older adults’ care in situations wherein implementing face-to-face services during the COVID-19 pandemic is difficult. We propose the development of non-face-to-face services and applying them to participants to evaluate their effectiveness through a comparison between experimental and control groups in future research.

## Figures and Tables

**Table 1:** Participants’ general characteristics (N = 100).

Variables		M ± SD/N (%)	Variables		M ± SD/N (%)
Age	Total	73.2 ± 7.0	Self-rated health status	Low	20 (20.0)
	≤70	49 (49.0)		High	15 (15.0)
	71~80	32 (32.0)		Middle	66 (66.0)
	≥81	19 (19.0)	Exercise implementation	Yes	71 (71.0)
Gender	Men	33 (33.0)		No	29 (29.0)
	Women	67 (67.0)	Types of exercise (multiple responses; *n* = 71)	Walking	58 (81.7)
Residence	Alone	22 (22.0)		Running	1 (1.4)
	With spouse	44 (44.0)		Swimming	3 (4.2)
	With spouse, sons and daughters	24 (24.0)		Hiking	2 (2.8)
	With sons and daughters	9 (9.0)		Gymnastics	11 (15.5)
	Others	1 (1.0)		Bicycle	7 (9.9)
Education	Middle school or lower	37 (37.0)		Others	10 (14.1)
	High school or higher	63 (63.0)	Experience in using smart device	Yes	52 (52.0)
Self-rated economic status	High	13 (13.0)		No	48 (48.0)
	Middle	67 (67.0)	Current medical history	Yes	88 (88.0)
				No	12 (12.0)

**Table 2:** Non-face-to-face services’ perception and necessity (N = 100).

Variables		N (%)
The need for non-face-to-face services for older adults	Strongly agree	7 (7.0)
	Agree	41 (41.0)
	Weakly agree	29 (29.0)
	Neither agree nor disagree	12 (12.0)
	Disagree	8 (8.0)
	Strongly disagree	3 (3.0)
Intention to use	Yes	72 (72.0)
	No	28 (28.0)
Number of interventions	Once a week	37 (37.0)
	Twice a week	38 (38.0)
	Three times a week	19 (19.0)
	Four times a week	1 (1.0)
	Five times a week	5 (5.0)
Time per episode	Less than 30 min	47 (47.0)
	More than 30 min to less than 1 h	44 (44.0)
	More than 1 h to less than 1 h 30 min	4 (4.0)
	More than 1 h 30 min to less than 2 h	3 (3.0)
	More than 2 h	2 (2.0)

**Table 3:** Preference for non-face-to-face service components (N = 100).

Contents	Details	M ± SD
Talking with smart devices	Conversation with smart devices through voice recognition and AI speaker functions	2.91 ± 1.22
Video calls	Personalized video calls for older adults to easily participate in social activities because they are friendly and easy to communicate with people who frequently talk on the phone	3.20 ± 1.19
Listening to music	Listening to music that can be easily accessed and manipulated by securing preferred music sources	3.37 ± 1.15
Sharing pictures	Sharing pictures by accessing a community hub through smart devices	3.41 ± 1.25
Reading books	Reading books through smart devices reflecting the results of the preferred book genre survey	3.24 ± 1.25
Life information	Checking everyday information—such as weather and news—through smart devices	3.33 ± 1.29
Silver game	Playing games through smart devices reflecting the results of the preferred cognitive game genre survey	2.90 ± 1.33
Calendar and schedule	Checking one’s schedule—including anniversaries and meetings—through smart devices	3.05 ± 1.14
Medication alarm	Checking medication time alarm through smart devices	3.06 ± 1.29
Gymnastics with music	Connecting with smart devices to exercise with music in real-time classes	2.99 ± 1.28
Walking	Measuring steps per day through smart devices	3.27 ± 1.15
Health assessment	Measuring health information—such as oxygen saturation, pulse and blood pressure—through smart devices	3.67 ± 1.17
Nutrition education	Watching healthy eating and diet recipe videos through smart devices	3.10 ± 1.31
Daily intake record	Recording intake of water, fruits, vegetables, and meat through smart devices	2.78 ± 1.13

**Table 4:** Differences in preference related to non-face-to-face services according to age, gender, and education (N = 100).

Variables	Age					Gender				Education			
	≤70 (*n* = 49)	71~80 (*n* = 32)	≥81 (*n* = 19)	*p*	Post-Hoc	Men (*n* = 33)	Women (*n* = 67)	*t*	*p*	Middle school or lower (*n* = 37)	High school or higher (*n* = 63)	*t*	*p*
	Mean Rank	Mean Rank	Mean Rank			M ± SD	M ± SD			M ± SD	M ± SD		
Talking with smart devices	52.70	48.20	48.68	0.743		2.97 ± 1.16	2.88 ± 1.25	0.35	0.726	3.03 ± 1.30	2.84 ± 1.17	0.74	0.463
Video calls	50.31	49.32	50.32	0.987		3.03 ± 1.05	3.29 ± 1.25	−1.08	0.283	3.32 ± 1.23	3.13 ± 1.17	0.79	0.431
Listening to music	49.33	52.09	48.16	0.863		2.97 ± 1.10	3.58 ± 1.12	−2.56	0.013	3.54 ± 1.19	3.27 ± 1.12	1.12	0.266
Sharing pictures	51.39	51.50	46.53	0.793		2.85 ± 1.06	3.69 ± 1.25	−3.50	0.001	3.54 ± 1.26	3.33 ± 1.24	0.80	0.426
Reading books	51.85	51.02	43.58	0.532		2.97 ± 1.19	3.38 ± 1.26	−1.58	0.118	3.17 ± 1.34	3.29 ± 1.20	−0.46	0.650
Life information	48.40	52.63	52.34	0.766		3.52 ± 1.15	3.24 ± 1.35	1.07	0.290	3.32 ± 1.27	3.33 ± 1.31	−0.03	0.973
Silver game	53.56	47.77	47.21	0.570		3.21 ± 1.19	2.75 ± 1.37	1.74	0.085	2.73 ± 1.33	3.00 ± 1.33	−0.98	0.329
Calendar and schedule	49.04	51.34	52.84	0.862		3.18 ± 1.01	2.99 ± 1.20	0.86	0.394	3.08 ± 1.12	3.03 ± 1.16	0.21	0.836
Medication alarm	44.73	53.53	58.06	0.156		3.00 ± 1.30	3.09 ± 1.30	−0.32	0.749	3.19 ± 1.29	2.98 ± 1.30	0.76	0.447
Gymnastics with music	47.79	57.36	42.94	0.160		2.79 ± 1.02	3.09 ± 1.39	−1.23	0.223	2.89 ± 1.37	3.05 ± 1.23	−0.59	0.559
Walking	52.46	47.38	50.71	0.721		2.76 ± 1.15	2.61 ± 1.21	0.59	0.559	2.57 ± 1.26	2.71 ± 1.14	−0.60	0.552
Health assessment	44.73	60.34	48.79	0.045	a < b	3.42 ± 1.06	3.79 ± 1.21	−1.55	0.126	3.57 ± 1.09	3.73 ± 1.22	−0.67	0.506
Nutrition education	54.37	50.86	39.92	0.163		3.06 ± 1.03	3.12 ± 1.43	−0.23	0.815	2.73 ± 1.28	3.32 ± 1.28	−2.21	0.029
Daily intake record	52.29	51.61	44.03	0.515		2.67 ± 0.99	2.84 ± 1.20	−0.75	0.457	2.54 ± 1.17	2.92 ± 1.10	−1.63	0.106

a ≤70, b = 71~80.

**Table 5:** Differences in preference related to non-face-to-face services according to smart device experience and exercise implementation (N = 100).

Variables	Smart Device Experience (*n* = 100)				Exercise Implementation (*n* = 99)				
	Yes (*n* = 53)	No (*n* = 47)	*t*	*p*	Yes (*n* = 70)	No (*n* = 29)	Mann-Whitney U	Z	*p*
	M ± SD	M ± SD			M ± SD	M ± SD			
Talking with smart devices	2.98 ± 1.20	2.83 ± 1.24	0.62	0.537	2.87 ± 1.17	3.07 ± 1.31	878.00	−1.09	0.276
Video calls	3.31 ± 1.29	3.09 ± 1.06	0.94	0.349	3.12 ± 1.25	3.45 ± 0.99	837.00	−1.31	0.190
Listening to music	3.51 ± 1.23	3.22 ± 1.03	1.27	0.208	3.26 ± 1.18	3.64 ± 1.06	778.50	−1.64	0.101
Sharing pictures	3.49 ± 1.31	3.32 ± 1.18	0.68	0.496	3.50 ± 1.25	3.28 ± 1.19	907.50	−0.85	0.396
Reading books	3.19 ± 1.32	3.30 ± 1.17	−0.46	0.647	3.28 ± 1.28	3.21 ± 1.18	976.50	−0.19	0.847
Life information	3.60 ± 1.26	3.02 ± 1.26	2.31	0.023	3.46 ± 1.26	3.10 ± 1.29	848.00	−1.32	0.187
Silver game	3.02 ± 1.32	2.77 ± 1.34	0.95	0.345	2.89 ± 1.39	2.90 ± 1.21	1003.50	−0.09	0.928
Calendar and schedule	3.25 ± 1.22	2.83 ± 1.01	1.84	0.069	3.20 ± 1.15	2.72 ± 1.07	775.00	−1.92	0.055
Medication alarm	3.26 ± 1.39	2.83 ± 1.14	1.70	0.093	3.12 ± 1.33	2.97 ± 1.21	931.50	−0.55	0.582
Gymnastics with music	3.19 ± 1.35	2.76 ± 1.18	1.67	0.098	3.04 ± 1.33	2.90 ± 1.18	945.00	−0.44	0.657
Walking	3.56 ± 1.13	2.96 ± 1.10	2.67	0.009	3.36 ± 1.18	3.03 ± 1.09	838.50	−1.32	0.188
Health assessment	3.92 ± 1.22	3.38 ± 1.05	2.36	0.020	3.80 ± 1.23	3.41 ± 0.95	792.00	−1.79	0.073
Nutrition education	3.30 ± 1.26	2.87 ± 1.33	1.66	0.101	3.34 ± 1.30	2.59 ± 1.15	701.00	−2.49	0.013
Daily intake record	3.04 ± 1.13	2.49 ± 1.08	2.48	0.015	2.96 ± 1.17	2.38 ± 0.94	708.00	−2.51	0.012

**Table 6:** Differences in preference related to non-face-to-face services according to self-rated health and economic status (N = 100).

Variables	Self-Rated Health Status (*n* = 100)				Self-Rated Economic Status (*n* = 100)			
	High (*n* = 15)	Middle (*n* = 66)	Low (*n* = 19)	*p*	High (*n* = 13)	Middle (*n* = 67)	Low (*n* = 20)	*p*
	Mean Rank	Mean Rank	Mean Rank		Mean Rank	Mean Rank	Mean Rank	
Talking with smart devices	50.60	50.06	51.95	0.967	48.42	50.25	52.70	0.905
Video calls	48.60	48.18	57.32	0.444	61.65	50.00	42.43	0.153
Listening to music	38.10	54.05	45.53	0.098	58.96	46.07	57.74	0.123
Sharing pictures	37.53	50.70	60.03	0.070	56.23	51.33	44.00	0.438
Reading books	40.63	52.26	49.66	0.346	39.27	53.57	45.20	0.166
Life information	52.20	50.50	49.16	0.953	60.27	50.21	45.13	0.319
Silver game	58.97	51.30	41.03	0.173	48.00	51.61	48.40	0.855
Calendar and schedule	52.30	51.12	46.92	0.816	64.73	47.81	50.25	0.136
Medication alarm	47.40	50.26	51.22	0.919	57.62	49.76	45.85	0.496
Gymnastics with music	45.90	51.99	46.11	0.605	53.69	49.67	48.68	0.869
Walking	47.90	50.42	50.21	0.949	53.08	46.70	58.88	0.203
Health assessment	51.10	51.57	46.32	0.765	45.50	50.96	52.20	0.773
Nutrition education	41.83	52.78	49.42	0.389	58.54	49.99	46.98	0.496
Daily intake record	50.20	52.77	42.84	0.378	61.15	46.71	56.28	0.125

## Data Availability

The data that support the findings of this study are available from the corresponding author, upon reasonable request.

## Appendix A. Developed Questionnaire on Non-Face-to-Face Service Needs of Community-Dwelling Older Adults in South Korea

Questionnaire on Non-face-to-face service needs of community-dwelling older adults in South Korea

[Consent]

Welcome. Thank you very much for participating. This survey is conducted for the purpose of providing the most necessary services by surveying the needs for non-face-to-face services for older adults. Please check the appropriate boxes for your personal information, awareness of non-face-to-face services, needs, and components. The survey takes about 15 to 20 min to complete.

Your participation is voluntary. The survey results will not be used for any purpose other than this study. Your responses will remain confidential. If you agree to the study, please sign and answer this survey. Thank you very much for participating in the survey.

### Appendix A.1. Demographics

1. What is your age? (  )
2. What is your gender?

① Man	② woman

3. What is your residence status?

① Living alone	② With spouse	③ With spouse, sons, and daughters
④ With sons and daughters	⑤ Others ( )	

4. What is the highest level of education?

① No formal education	② Elementary school	③ Middle school
④ High school	⑤ College/University	⑥ Graduate school

5. What is your self-rated economic status?

① High	② Middle	③ Low

6. What is your self-rated health status?

① High	② Middle	③ Low

7. Are you currently diagnosed with any disease? (Multiple answers allowed)

① Hypertension	② Diabetes mellitus	③ Dementia
④ Depression	⑤ Respiratory disease	⑥ Parkinson’s disease
⑦ Arthritis	⑧ Osteoporosis	⑨ Cardiovascular disease
➉ Peripheral Neuropathy	⑪ Tuberculosis	⑫ Stroke
⑬ Arrhythmia	⑭ Other nervous system	⑮ Others ( )

8. Do you usually exercise?

① Yes (→ Go to Q9)	② No (→ Go to Q10)

9. (① for Q8) What kind of exercise do you mainly do?

① Walking	② Running	③ Swimming
④ Hiking	⑤ Gymnastics	⑥ Bicycle
⑦ Others ( )		

### Appendix A.2. Perception and Necessity of Non-Face-to-Face Services for Older Adults

※ **Definition of “non-face-to-face service” for older adults** The non-face-to-face service for the elderly is to mediate the health, safety, and emotional parts of the elderly with electronic devices such as smartphones and tablets without meeting people

10. Have you heard of non-face-to-face service?

① Yes	② No

11. Do you think non-face-to-face services are necessary for community-dwelling older adults?

① Strongly Agree		② Agree	③ Weakly agree
④ Neither agree nor disagree		⑤ Disagree	⑥ Strongly disagree
※	①–③ (→ Go to Q12)	⑤, ⑥ (→ Go to Q13)	

12. (①–③ for Q11) Why did you think the service was necessary?

① Because the elderly are inconvenient to move
② Because there is a risk of infection such as COVID-19
③ Smart programs for the elderly are increasing
④ I think it would be fun to use the service
⑤ Using the service seems to reduce loneliness
⑥ Others ( )

13. (⑤, ⑥ for Q11) Why did you think the service was unnecessary?

① It seems difficult to learn a new device
② It seems that it will cost a lot to buy the device
③ I want to use the device, but there is no one to help me
④ It is better to meet people in person and do activities
⑤ Others ( )

14. Will you participate in non-face-to-face service once it is established?

① Yes (→ go to Q15)	② No (→ go to Q16)

15. (“Yes” for Q14) What is the reason for participating the service?

① I think it will be interesting to experience new devices and services
② Difficulty in direct visits to public health centers, senior citizens’ centers, etc. due to difficulty in mobility
③ I think it would be better to have mediation at home because of COVID-19
④ I think I can get a lot of information about my health
⑤ Using the service seems to reduce loneliness
⑥ Others ( )

16. (“No” for Q14) What is the reason for not participating the service?

① No economic leeway
② It is better to visit and receive service in person
③ Inconvenient to use the device without a guardian
④ Fear of learning a new device
⑤ Wi-Fi is not available
⑥ Others ( )

17. How much participation do you think is appropriate for non-face-to-face service?

① Once a week	② Twice a week	③ Three times a week
④ Four times a week	⑤ Five times a week	

18. How many hours do you think is appropriate for non-face-to-face service?

① Less than 30 min	② More than 30 min to less than 1 h	③ More than 1 h to less than 1 h 30 min
④ More than 1 h 30 min to less than 2 h	⑤ More than 2 h	

19. What is your favorite music genre? (Multiple answers allowed)

① Trot	② Popular song	③ Classic
④ Traditional music	⑤ Hymn	⑥ Others ( )

20. What is your favorite game? (Multiple answers allowed)

① Shogi	② Hwatu	③ Omok
④ Jigsaw puzzle	⑤ Pairing	⑥ Playing cards
⑦ Memory quiz	⑧ Baduk	⑨ Smart game
➉ Others ( )		

21. What is your favorite book genre? (Multiple answers allowed)

① Traditional stories	② Fairytale	③ Poem
④ Novel	⑤ Religious books	⑥ Others ( )

22. What is fun activities? (Multiple answers allowed)

① Biking	② Climbing	③ Walking
④ Gymnastics	⑤ Running	⑥ Terra band
⑦ Golf	⑧ Ping-pong	⑨ Badminton
➉ Dance	⑪ Others ( )	

### Appendix A.3. The Preference of Non-Face-to-Face Services for Older Adults

Please rate the most preference of each of the following components of non-face-to-face services for older adults.

**Component**		**Preference**				
		**Not at All Preferred**	**Low Preferred**	**Moderate**	**Preferred**	**Very Preferred**
		**1**	**2**	**3**	**4**	**5**
23. Taking with smart devices						
24. Video calls						
25. Listening to music						
26. Sharing pictures						
27. Reading books						
28. Life information (Weather, news, etc.)						
29. Silver game						
30. Calendar and schedule						
31. Medication alarm						
32. Gymnastics with music						
33. Walking						
34. Health assessment (Oxygen saturation, pulse, blood pressure, blood sugar, body composition test, etc.)						
35. Nutrition education						
36. Daily intake record (Water, fruits, and vegetables, meat)						
Comments						
